# Cost-effectiveness of maternal influenza immunization in Bamako, Mali: A decision analysis

**DOI:** 10.1371/journal.pone.0171499

**Published:** 2017-02-07

**Authors:** Evan W. Orenstein, Lauren A. V. Orenstein, Kounandji Diarra, Mahamane Djiteye, Diakaridia Sidibé, Fadima C. Haidara, Moussa F. Doumbia, Fatoumata Diallo, Flanon Coulibaly, Adama M. Keita, Uma Onwuchekwa, Ibrahima Teguete, Milagritos D. Tapia, Samba O. Sow, Myron M. Levine, Richard Rheingans

**Affiliations:** 1 Emory University School of Medicine, Atlanta, Georgia, United States of America; 2 Le Centre pour le Développement des Vaccins du Mali (CVD-Mali), Bamako, Mali; 3 Department of Obstetrics and Gynecology, Gabriel Touré Teaching Hospital, Bamako, Mali; 4 Center for Vaccine Development, University of Maryland School of Medicine, Baltimore, Maryland, United States of America; 5 Department of Environmental & Global Health, University of Florida, Gainesville, Florida, United States of America; Imperial College London, UNITED KINGDOM

## Abstract

**Background:**

Maternal influenza immunization has gained traction as a strategy to diminish maternal and neonatal mortality. However, efforts to vaccinate pregnant women against influenza in developing countries will require substantial investment. We present cost-effectiveness estimates of maternal influenza immunization based on clinical trial data from Bamako, Mali.

**Methods:**

We parameterized a decision-tree model using prospectively collected trial data on influenza incidence, vaccine efficacy, and direct and indirect influenza-related healthcare expenditures. Since clinical trial participants likely had better access to care than the general Malian population, we also simulated scenarios with poor access to care, including decreased healthcare resource utilization and worse influenza-related outcomes.

**Results:**

Under base-case assumptions, a maternal influenza immunization program in Mali would cost $857 (95% UI: $188-$2358) per disability-adjusted life year (DALY) saved. Adjusting for poor access to care yielded a cost-effectiveness ratio of $486 (95% UI: $105-$1425) per DALY saved. Cost-effectiveness ratios were most sensitive to changes in the cost of a maternal vaccination program and to the proportion of laboratory-confirmed influenza among infants warranting hospitalization. Mean cost-effectiveness estimates fell below Mali’s GDP per capita when the cost per pregnant woman vaccinated was $1.00 or less with no adjustment for access to care or $1.67 for those with poor access to care. Healthcare expenditures for lab-confirmed influenza were not significantly different than the cost of influenza-like illness.

**Conclusions:**

Maternal influenza immunization in Mali would be cost-effective in most settings if vaccine can be obtained, managed, and administered for ≤$1.00 per pregnant woman.

## Introduction

Maternal immunization has emerged as a potential strategy to mitigate maternal and neonatal mortality. In addition to protecting the pregnant mother, maternal vaccination may protect the fetus and infant in the critical first months of life through transfer of IgG antibodies across the placenta [1]. In high-income countries, vaccination against tetanus, influenza, hepatitis B, and invasive meningococcal disease is recommended in pregnant women [2]. While maternal tetanus vaccination has been shown to be cost-effective [3] and has cut the rates of neonatal tetanus in half in low-income countries [4], adoption of other maternal vaccines has lagged. Interest in maternal influenza immunization in developing countries is growing, with recently completed randomized-controlled trials in Nepal [NCT01034254], Mali [NCT01430689], and South Africa [NCT01306669] [5,6].

The risk of complications from influenza infection is significantly higher in pregnant women [7] and infants <6 months [8], and the latter are precluded from immunization with currently licensed vaccines. Influenza vaccine during pregnancy has been shown to be safe [9] and cost-effective in high-income countries [10–12]. Randomized controlled trials of maternal influenza vaccine in Bangladesh, South Africa, and Mali found 63%, 50%, and 70% fewer episodes of laboratory-confirmed influenza (LCI) in infants of mothers vaccinated against influenza compared to infants of mothers vaccinated against other illnesses [6,13,14]. The additional impact on young infants suggests that maternal influenza vaccine may be cost-effective in low-income countries.

Adoption of maternal influenza immunization programs in low-income countries will require a firm case for investment. The cost-effectiveness ratio (CER) will depend on the health benefits of vaccination including decreased influenza-related morbidity and mortality for mothers and their infants, the economic benefits of vaccination averting influenza-related healthcare expenditures, and the programmatic costs of vaccination including supplies as well as the infrastructure to manage and administer influenza vaccine to pregnant women. We collected prospective data on direct and indirect costs of laboratory confirmed influenza (LCI) and influenza-like illness (ILI) incurred during the trial in Mali. We combined these results with epidemiological and vaccine efficacy data [14] to parameterize a decision-tree model of the cost-effectiveness of maternal influenza immunization in Mali.

## Methods

### Model structure

**Fig 1:**
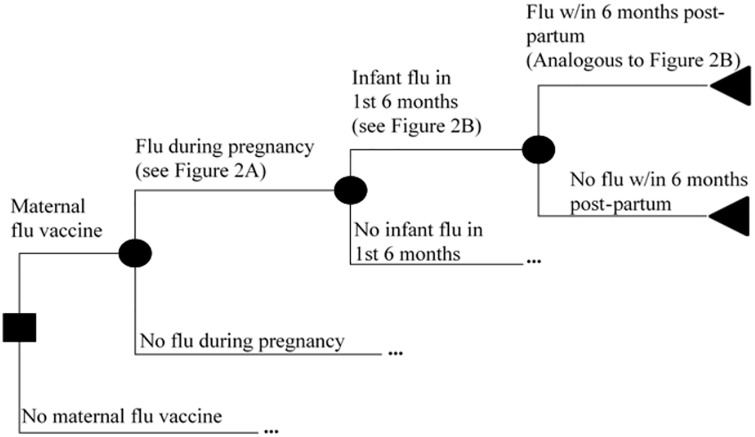
Overall decision-tree model structure. Squares designate decision points. Circles designate probabilistic events. Triangles designate terminal nodes. Ellipses (…) indicate a symmetrical sub-tree that is not shown due to space constraints.

**Fig 2:**
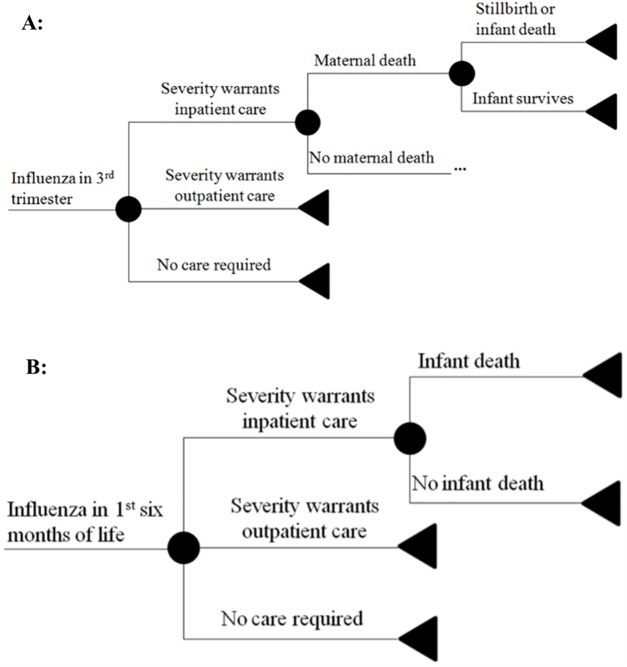
Influenza sub-tree structures. **(A)** Series of events that determine the monetary and utility costs of influenza infection during pregnancy. (**B)** Series of events that determine the monetary and utility costs of influenza infection during the first 6 months of life.

We built a decision tree model of the costs and benefits of maternal influenza immunization. All benefits of maternal influenza vaccine were assumed to stem from prevention of laboratory-confirmed influenza in the pregnant mother, the infant, or the post-partum mother. After an initial decision to either vaccinate or not vaccinate the pregnant mother, further events including influenza infection in the pregnant woman, infant, or post-partum mother proceeded in a probabilistic manner (Fig 1). At each node of influenza infection, a sub-tree determined the associated monetary costs from treatment and the loss of disability-adjusted life years (DALYs) (Fig 2). Each infection was stratified by severity as requiring no treatment, outpatient therapy only, or inpatient therapy. Healthcare encounters including influenza requiring outpatient or inpatient therapy were each associated with monetary costs of illness. The outcomes of maternal death, stillbirth, and infant death each resulted in a loss of DALYs as a function of the life expectancy at the time of the event [15], with maternal death estimated at 24.7 years (the average age at enrollment) [14]. All branches were identical in the vaccine and no-vaccine branches except in the probability of contracting influenza. The incremental cost-effectiveness of maternal influenza immunization was calculated by dividing the difference in net costs in the vaccine and no-vaccine branches by the difference in DALYs lost between the two branches.

All calculations were based on a societal perspective. To reduce the likelihood of overestimating the cost-effectiveness of maternal influenza immunization, we only included DALY losses from premature death [15] and costs related to the acute episode; long-term sequelae were excluded from the model. Thus, we did not specify an analytical horizon, discount rate, or base year for dollars.

### Parameterization

Attack rates of influenza in mothers and infants, vaccine efficacy in mothers and infants, the risk of infant hospitalization after contracting LCI, and outpatient and inpatient costs of illness for mothers and infants were all taken from primary data collected during a phase 4 randomized controlled effectiveness trial conducted in Bamako, Mali from September 2011 –January 2014 that determined the efficacy, safety, and immunogenicity of trivalent inactive influenza vaccine (TIV) in pregnant women and their infants up to 6 months of age. Full study details are described elsewhere[14] but are summarized below with an emphasis on collection of cost data. Additional parameters for which the trial in Mali was underpowered were obtained through literature review (Table 1). Methods of estimation for all parameters are given in detail in the Supplementary Information (S1 Table). The threshold for cost-effectiveness was set to the 2013 gross domestic product (GDP) per capita in Mali, US$715 [14].

**Table 1:** Baseline parameters and ranges used in the model.

Parameter	Baseline Value	Range	Distribution	References
*Burden of influenza illness*				
Attack rate (mothers)	0.010	0.006–0.015	Triangular	[14]
Attack rate (infants)	0.028	0.022–0.037	Triangular	[14]
Risk of hospitalization given influenza (pregnant women)	0.006	0.004–0.010	Triangular	[27–29]
Risk of hospitalization given influenza (post-partum women)	0.002	0.001–0.004	Triangular	[27]
Risk of hospitalization given influenza (infants)	0.013	0.0003–0.070	Triangular	[14]
CFR of influenza-attributable hospitalization (pregnant women)	0.080	0.067–0.094	Triangular	[30]
CFR of influenza-attributable hospitalization (post-partum women)	0	N/A	Point estimate	[31]
CFR of influenza-attributable hospitalization (infants)	0.044	0.012–0.077	Triangular	[20,32]
Additional risk of stillbirth or neonatal death given pregnant mother hospitalized due to influenza	0.036	0.015–0.088	Triangular	[33,34]
*Vaccine efficacy*				
Mothers	70.3%	42.2%– 85.8%	Triangular	[14]
Infants in 1^st^ 5 months of life	60.7%	33.8%– 77.5%	Triangular	[14]
*Costs*				
Influenza vaccine (per pregnant woman)	$1	$0.50 –$2.00	Triangular	[3]
Influenza-attributable hospitalization (mother)	$157.51	$34.39 –$280.62	Triangular	Table 2
Influenza-attributable hospitalization (infant)	$157.50	$131.20 –$189.09	Triangular	Table 2
Influenza-attributable outpatient visit (mother)	$4.83	$3.72 –$6.27	Triangular	Table 2
Influenza-attributable outpatient visit (infant)	$4.41	$3.99 –$4.87	Triangular	Table 2
Programmatic cost per pregnant woman vaccinated	$1.00	$0.50-$2.00	Triangular	
*Utilities*				
DALYs lost for maternal death	32.27	---	Point estimate	[14,35]
DALYs lost for infant death or stillbirth	57.28	---	Point estimate	[35]
*Access to Care*				
Proportion with inadequate access to care	0.604	0.507–0.730	Triangular	[16]
Relative risk of death without access to care	3	1–5	Triangular	Assumption

CFR = Case-fatality ratio; LBW = low birth weight; DALY = Disability-adjusted life year.

### Primary data collection and analysis

Pregnant women were recruited during their 3^rd^ trimester at 6 community and referral health centers in Bamako, Mali. After randomization, women were vaccinated with either TIV or meningococcal conjugate vaccine (MCV). From enrollment until the infant reached age 6 months, field personnel performed weekly visits, during which the participating woman and infant (if already born) had their temperatures measured and were evaluated for ILI. When the ILI case definition was met nasopharyngeal/oropharyngeal swabs and a malaria blood smear were obtained, and a team dedicated to estimating costs of illness was contacted. For participants treated as outpatients, study personnel visited the home every 2–5 days for the duration of the episode to ascertain the direct costs of illness (including medications, labs, traditional healers, and transportation) and indirect costs of illness (defined as the number of workdays lost by each family member multiplied by that family member’s average daily earnings). Additionally, all medications prescribed by study physicians and filled at the recruitment health centers were reimbursed by the study and counted as direct costs. Infants with severe ILI warranting hospitalization were admitted to l’Hôpital Gabriel Touré, a public university hospital, and were visited by a study physician daily who accounted for direct and indirect costs. In addition to direct costs to families that were reimbursed through the maternal influenza study, other diagnostic tests (complete blood count, blood cultures, chest radiographs) were provided free of charge to some patients through separate ongoing studies when indicated; the costs of these tests were added back into total cost estimates.

Inpatient episodes included all direct and indirect costs incurred in both the outpatient and inpatient setting for a single ILI episode and were modelled with a log-normal distribution. Outpatient episodes included illnesses with non-zero total costs but with no hospitalization; these were also modelled with a log-normal distribution. All episodes included zero and non-zero costs of outpatient and inpatient episodes and were modelled with an exponential distribution. Statistics were performed using Matlab Version 7.7 (The Mathworks, Inc).

Approval for the research was obtained from the University of Maryland, Baltimore Institutional Review Board; the ethics committee of the Faculté de Médecine, Pharmacie et Odonto-Stomatologie of Mali; and the Ministry of Health of Mali.

### Sensitivity analyses

We performed univariate and multivariate sensitivity analyses to ascertain the major determinants of the cost-effectiveness of maternal influenza immunization. First, each input variable was individually changed by a relative proportion to assess its impact on the CER. Second, we performed a regression tree analysis on 10,000 Monte Carlo simulations that randomly sampled all input variables across their distributions simultaneously. The regression tree algorithm found the threshold value of the single input variable that best divides all simulations into two groups, minimizing the variance in CERs within each group. This process yielded the single parameter and threshold value with the highest predictive power in estimating the CER. The algorithm then performed the same calculation on each respective branch to determine the next most important parameters in predicting the CER and repeated until the number of simulations in each leaf was <10. Finally, the tree was pruned to prevent over-fitting using a 10-fold cross-validation strategy that minimizes generalization error.

**Fig 3:**
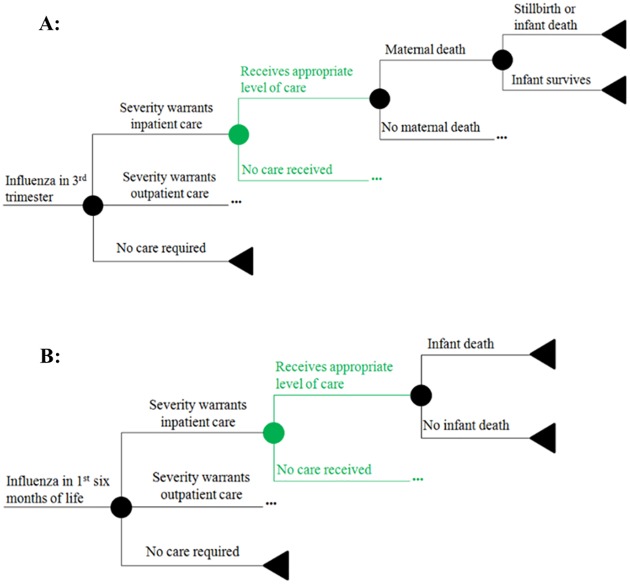
Changes to influenza sub-trees when accounting for decreased access to care. **(A)** Changes to the sub-tree for influenza infection during pregnancy. (**B)** Changes to the sub-tree for influenza infection during the first 6 months of life.

Under clinical trial conditions, active surveillance likely led to earlier detection of illness, higher healthcare resource utilization, and better health outcomes. For example, low birth weight incidence in the Mali trial was significantly lower than that found in Bamako in Demographic and Health Surveys (DHS) [14,16]. Thus, we modeled different levels of access to medical care (Fig 3) by varying the proportion of ill individuals who received care at the level warranted by disease severity (inpatient, outpatient, or no care required). The health outcomes and costs were adjusted according to whether the individual received the appropriate level of care; a person who did not receive adequate care had a higher likelihood of death but did not incur the costs of outpatient or inpatient illness.

Finally, we performed a two-way sensitivity analysis to look at the impact of changing attack rates on vaccination strategies. Season-to-season and geographical variability in influenza incidence may profoundly affect cost-effectiveness. Additionally, vaccinating earlier in pregnancy might provide greater protection throughout the pregnancy, but may result in less antibody transfer and protection of the infant. Conversely, vaccinating in the 3^rd^ trimester likely maximizes infant protection, but leaves the pregnant woman unimmunized for a larger proportion of the pregnancy. We therefore examined how the interaction of these two attack rates affected the CER.

The model was built and analyzed using the Treeplan, Sensit, and Risksim macros (Decision Support Services, San Francisco) in MS Excel as well as Matlab Version 7.7 (The Mathworks, Inc). We calculated 95% confidence intervals (CI) for primary cost data and 95% uncertainty intervals (UI) for Monte Carlo simulations.

## Results

### Cost of influenza-related illness

We captured costs for 188 of 193 laboratory-confirmed influenza (LCI) episodes (including 184 outpatient episodes, 1 inpatient episode, and 3 episodes with total cost $0.00) and 6,807 of 7,770 influenza-like illness (ILI) episodes (including 6,383 outpatient episodes, 63 inpatient episodes, and 361 episodes with total cost $0.00). The average total cost per case for 188 episodes of LCI across all populations was $5.84 (CI: $5.08-$6.77), compared to $5.50 (CI: $5.37-$5.63) for 6,807 ILI episodes (Table 2). There were no significant differences comparing cost of LCI to ILI or between infants and mothers. The average outpatient episode cost was $4.52 (CI: $4.09-$5.00) for 184 cases of LCI and was $4.19 ($4.12-$4.27) in 6,383 cases of ILI. Among 63 ILI episodes requiring hospitalization, the average cost was $157.50 (CI: $131.20-$189.09). Only 1 episode of LCI requiring hospitalization had complete cost data captured ($247.37). Of all costs, 97% were direct (45% from medicines and 43% from chest X-rays) and 3% indirect.

**Table 2:** Costs of laboratory-confirmed influenza (LCI) and influenza-like illness (ILI) episodes in stratified by population and vaccination status in the setting of a maternal influenza vaccine trial in Bamako, Mali.

Population	N	Model	Sample Mean	Mean estimate (95% CI)
*Laboratory Confirmed Influenza (LCI)*				
All episodes	188	Exponential	$5.84	$5.84 ($5.08 - $6.77)
TIV^x^	66		$4.35	$4.35 ($3.46 - $5.62)
MCV^¥^	122		$6.64	$6.64 ($5.60 - $8.00)
Outpatient*	184	Log-Normal	$4.62	$4.52 ($4.09 - $5.00)
TIV^x^	64		$4.48	$4.30 ($3.65 - $5.07)
MCV^¥^	120		$4.69	$4.65 ($4.09 - $5.28)
Infants	132	Log-Normal	$4.56	$4.41 ($3.99 - $4.87)
TIV^x^	53		$4.48	$3.99 ($3.45 - $4.62)
MCV^¥^	79		$4.86	$4.70 ($4.11 - $5.37)
Women	52	Log-Normal	$4.77	$4.83 ($3.72 - $6.27)
TIV^x^	11		$6.33	$6.37 ($3.02 - $13.46)
MCV^¥^	41		$4.36	$4.53 ($3.44 - $5.96)
Inpatient^†^	1 (1 infant; mother received MCV)	None	$247.37	---
*Influenza-like illness (ILI)*				
All episodes	6807	Exponential	$5.50	$5.50 ($5.37 - $5.63)
Outpatient*	6383	Log-Normal	$4.31	$4.19 ($4.12 - $4.27)
Infants	5057	Log-Normal	$4.34	$4.15 ($4.08 - $4.24)
Women	1326	Log-Normal	$4.21	$4.30 ($4.11 - $4.50)
Inpatient^†^	63 (61 infants, 2 pregnant women)	Log-Normal	$156.62	$157.50 ($131.20 - $189.09)
*Non-influenza illness (Non-LCI ILI)*				
All episodes	6619	Exponential	$5.49	$5.49 ($5.36 - $5.62)
Outpatient	6199	Log-Normal	$4.31	$4.18 ($4.10 - $4.26)
Infants	4925	Log-Normal	$4.34	$4.15 ($4.07 - $4.23)
Women	1274	Log-Normal	$4.19	$4.28 ($4.09 - $4.49)
Inpatient^†^	62	Log-Normal	$155.16	$155.78 ($129.58 - $187.28)

* Outpatient episodes include those where cost data were obtained, the cost of the episode was greater than $0.00, and the participant was never hospitalized during the course of their illness; by contrast “All episodes” includes 3 episodes where the total cost was $0.00 as well as the 1 episode where the infant was hospitalized.

^†^ Inpatient episodes include costs incurred in both the inpatient and outpatient setting associated with the ILI episode that required hospitalization.

^x^ TIV indicates episodes in households where the pregnant woman received trivalent influenza vaccine.

^¥^ MCV indicates episodes in households where the pregnant woman received meningococcal conjugate vaccine.

### Cost-effectiveness estimates

**Fig 4:**
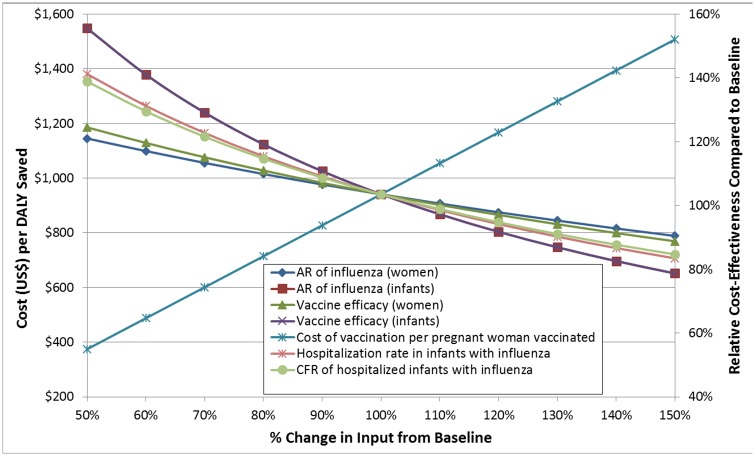
One-way sensitivity analysis of the base case. Each input variable is evaluated on a separate curve. The X-axis shows the percent change in the input variable from its baseline value (set at 100%), and the Y-axis shows the cost per DALY saved (left) and the relative change in the cost-effectiveness ratio from its initial value (right). Moving down in the Y-axis indicates a lower cost per DALY, i.e. a more efficient intervention. The greater the slope of each curve, the more sensitive the baseline model is to changes in that variable. The 7 most sensitive variables are depicted here.

**Fig 5:**
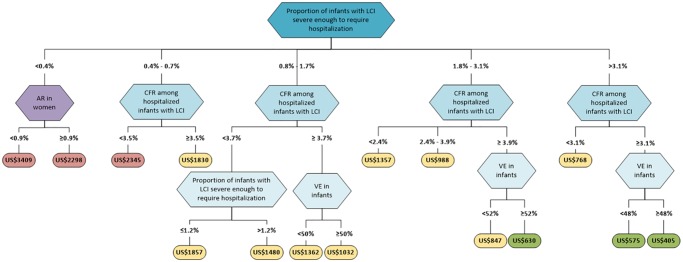
Regression tree analysis of 10,000 Monte Carlo simulations sampling across the uncertainty in all parameters. The leaves of the tree end in ovals that show the mean cost per DALY of maternal influenza vaccine among all simulations whose parameters follow the paths described in the proximal branches. Further partitioning of this pruned, cross-validated tree did not reduce generalization error. Parameters affecting infant influenza are shown in shades of blue, while parameters affecting influenza in women are shown in purple. Cost-effectiveness ratios < 1x per-capita GDP in Mali are shown in green, those between 1x and 3x per-capita GDP in Mali are shown in yellow, those >3x per-capita GDP in Mali are shown in red. LCI: Lab-confirmed influenza; CFR: case-fatality ratio; VE: vaccine efficacy.

Under baseline assumptions, the cost-effectiveness of maternal influenza immunization in Mali was estimated at $857 (UI: $188-$2358) per disability-adjusted life year (DALY) saved (Table 3). Adjusting for poor access to care resulted in a cost-effectiveness ratio (CER) of $486 (95% UI: $105-$1425) per DALY saved. In univariate sensitivity analyses, the cost of the vaccination program per vaccinated woman, attack rate of influenza in infants, and vaccine efficacy in preventing infant influenza had the greatest impact on CER (Fig 4). Generally, parameters affecting the frequency and severity of infant influenza had higher impact than parameters affecting influenza in mothers (Supplementary Material, S1 Fig). In regression tree analysis, the hospitalization rate of infants with influenza and case-fatality ratio (CFR) among hospitalized infants were the most important determinants of cost-effectiveness (Fig 5). Of note, these two parameters had relatively high uncertainty compared to outcomes for which the clinical trial was powered to assess and may vary between populations and between influenza seasons. Based on these analyses, we also examined a “severe disease” scenario, in which the hospitalization rate for infants was elevated to the high end of uncertainty, yielding $311 (UI: $86-$749) per DALY saved or $184 (UI: $58-$458) with additional adjustment for poor access to care.

**Table 3:** Changes in the cost-effectiveness ratio of maternal influenza immunization from the baseline from limited access to healthcare, changing the attack rate of influenza and the vaccine efficacy to rates reported in other randomized trials, and adjusting the costs of illness based on per-capita healthcare spending.

Scenario	Model Changes	Cost per DALY (95% CI)
**Baseline**	None	$857 (UI: $188 –$2358)
**Poor access to care**	Decreased access to care (Fig 3)	$486 (UI: $105 –$1425)
**Severe Disease**	Risk of hospitalization for infants with LCI set to upper limit 7.0%	$311 (UI: $86 - $749)
**Severe Disease & Poor access to care**	Decreased access to care (Fig 3) Risk of hospitalization for infants with LCI set to upper limit 7.0%	$184 (UI: $58-$458)

AR = Attack rate; VE = Vaccine efficacy; DALY = disability-adjusted life year; UI: Uncertainty interval.

### Impact of programmatic costs

**Fig 6:**
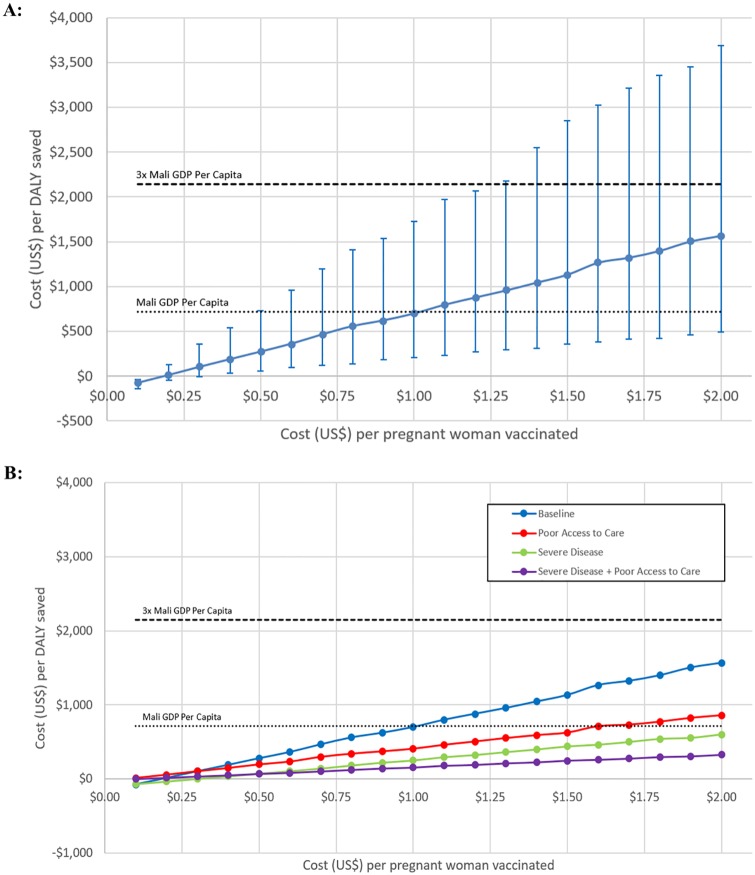
Cost (US$) per DALY saved of a maternal influenza immunization program varied by cost of vaccination program per pregnant woman vaccinated. **(A)** Baseline model. **(B)** Comparison across 4 scenarios: Baseline, Poor Access to Care, Severe Disease, and Severe Disease + Poor Access to Care.

**Fig 7:**
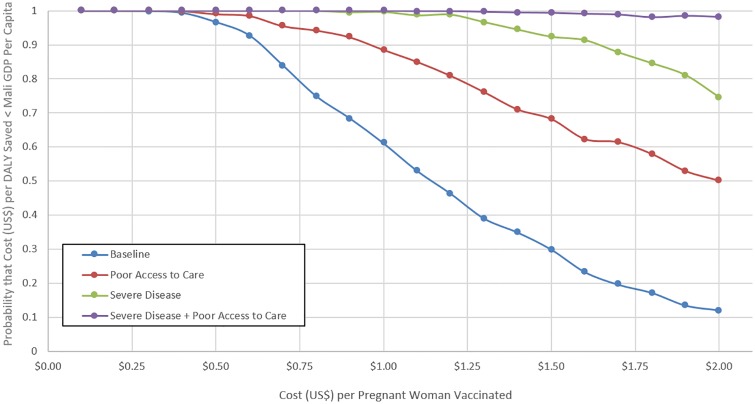
Effect of the cost of a maternal immunization program on the probability that the program will be cost-effective. Probability that the cost per DALY saved < Mali GDP per capita by programmatic costs per pregnant woman vaccinated across 4 scenarios: Baseline, Poor Access to Care, Severe Disease, and Severe Disease + Poor Access to Care.

Given the high sensitivity of our model to programmatic costs of vaccinating pregnant women and the reality of changing prices for vaccine itself, supplies, personnel, fuel, and other administrative costs, we calculated estimates of expected cost-effectiveness at different total price points for vaccinating pregnant women (Fig 6) and the probability that the cost per DALY saved was less than Mali gross domestic product (GDP) per capita (Fig 7). At $0.50 total cost per pregnant woman vaccinated in the baseline model, cost-effectiveness estimates ($271.49, UI: $53.39-$711.64) were significantly lower than Mali GDP per capita. Mean cost-effectiveness estimates exceed Mali GDP per capita at $1.00 per pregnant woman vaccinated. When adjusting for access to care, cost-effectiveness was significantly lower than Mali GDP per capita up to $0.67 per vaccinated pregnant woman, and mean estimates exceeded this threshold at $1.65. With severe disease and adjustment for access to care, cost-effectiveness estimates were significantly lower than Mali GDP per capita up to $2.00 per vaccinated pregnant woman.

### Changing attack rates

**Fig 8:**
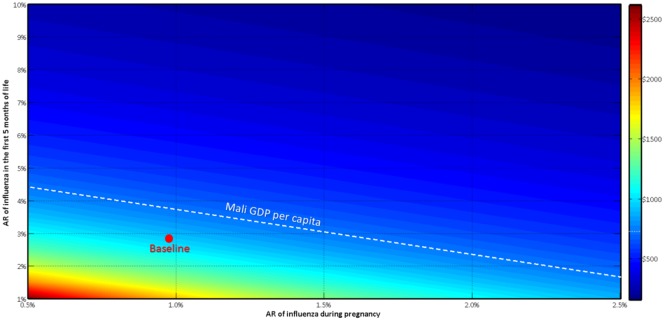
Impact of changing attack rates of influenza during pregnancy and in the first 5 months of life on the cost-effectiveness of maternal influenza immunization. AR: Attack Rate.

We studied the interaction of the attack rates of influenza in pregnant women and infants (Fig 8). Perturbations in the attack rate in infants had greater impact on the cost per DALY than similar changes in the attack rate in pregnant women. If the attack rate doubled in infants but was cut in half in women the cost per DALY decreased from $857 to $511.62 (UI: $65.21-$1833.47). By contrast, doubling the attack rate in pregnant women while decreasing the attack rate in infants by 50% led to slight increase in cost per DALY to $961.01 (UI: $289.80-$2239.97).

## Discussion

Maternal influenza immunization has the potential to be a high-impact, cost-effective intervention for reducing maternal and neonatal morbidity and mortality in low-income countries if programmatic costs are sufficiently low. Cost-effectiveness estimates were significantly lower than per capita GDP in Mali when total cost per vaccinated pregnant woman was less than $0.50, and mean estimates were lower than this threshold at a cost of $1.00 per vaccinated pregnant woman. Maternal immunization’s impact on infant influenza burden provided the greatest benefits in DALYs saved. Cost-effectiveness was most improved in scenarios with increased infant attack rates, hospitalization rates, CFR, and vaccine efficacy.

Policy makers seeking to implement maternal influenza vaccine in low-income settings will confront many challenges including deciding how to focus resources on vaccine programs. Efforts aimed at more remote areas, while more costly to administer, would likely yield higher mortality benefits; in populations with poor access to care, mean CER estimates were lower than per capita GDP in Mali at total cost of $1.67 per pregnant woman vaccinated. Logistical considerations such as combination with maternal tetanus vaccine programs or in campaigns for seasonal illnesses in low-income countries (e.g. meningococcus) may also play an important role in the most appropriate timing and location of maternal influenza vaccine programs.

To our knowledge, no previous studies have estimated the costs of influenza illness in sub-Saharan Africa [17]. One other study [18] estimated the costs of influenza illness in a low-income country, finding similar median costs of outpatient influenza ($4.80 per episode) and influenza-related hospitalization (US$82.20); however, no formal model of the cost-effectiveness of maternal influenza immunization was pursued. Previous models have focused on developed countries, including the United States [10,12] and the United Kingdom [11], with CERs ranging from $37,000 to $70,089 per quality-adjusted life year. Our CER was dramatically lower, reflecting lower costs of healthcare utilization and worse health outcomes attributable to influenza in low-resource settings.

As in all mathematical models, the conclusions from this study are limited by the quality of data informing the parameters. Many parameters including attack rates of influenza, vaccine efficacy, hospitalization rates in infants, and economic impact of influenza are based on two years of primary data collected during a clinical trial in Mali. However, geographic and seasonal variation in influenza severity, vaccine match, and market forces may change these key parameters substantially [19]. Additionally, most DALYs saved in our model stemmed from prevention of infant mortality. As the trial was underpowered to assess influenza mortality and ethical considerations led to study team interventions that likely interfered with the natural progression of influenza severity, we relied on meta-estimates from studies of CFR of severe influenza infection in other developing countries, but only 1 of these 10 studies came from Sub-Saharan Africa [20]. Finally, while DHS surveys in Mali estimate the proportion of Bamako residents who did not seek care for acute illness [16], the increased morbidity and mortality from this poor access is nearly impossible to assess, requiring assumptions from the authors to model this important phenomenon.

While all of these factors decrease the generalizability of this model to future influenza seasons and different low-income countries, we also did not account for several factors that may increase the benefit of maternal influenza vaccine including decreased influenza severity in vaccinated mothers and infants [21–23], maternal HIV infection [6], and herd immunity. We also did not include possible improvement of birth weight through maternal influenza immunization [24,25], since no evidence for this effect was found in the Mali trial, although this would substantially improve cost-effectiveness.

Maternal immunization holds promise as a key strategy in the reduction of neonatal and infant mortality through immunologic mechanisms that protect vulnerable young infants too immature to respond effectively to direct vaccination. A maternal influenza immunization program in Mali would likely be highly cost-effective with cost per DALY saved significantly less than per capita GDP if the total cost of the vaccination program could be kept less than $0.50 per pregnant woman vaccinated. Supplemental immunization activities for maternal tetanus vaccine in Pakistan achieved costs as low as $0.40 per dose [3], and coupling influenza vaccine with maternal tetanus vaccine may result in even lower overall programmatic costs. Additionally, in years of seasonal influenza epidemics, vaccine manufacturing companies may have excess vaccine that could be diverted to low-income countries [26]. When comparing the CER of maternal influenza immunization to competing priorities in low-resource settings, circumspect policy makers in low-resource countries must weigh not only the public health impact and purported CER of an intervention, but also the strength of the data and direction of potential biases in CER calculations. Our study is based on prospectively collected clinical and cost data and makes consistently conservative assumptions biasing towards a higher CER. Nonetheless, we show that investment in maternal influenza immunization is highly cost-effective from a global societal perspective. The case for investment is most compelling in sub-populations with higher influenza attack rates and poor access to care. Further surveillance examining influenza incidence and severity across time and space, particularly in young infants, will determine in which settings to pursue maternal influenza immunization programs.

## Supporting information

S1 Fig Complete one-way sensitivity analysis of the base case model. (TIF)

S1 Table Complete parameter list with explanations. (DOCX)
