# Supplementary figures and images for: Cost-effectiveness of maternal influenza immunization in Bamako, Mali: A decision analysis
Source: PLoS One. 2017 Feb 7;12(2):e0171499. doi: 10.1371/journal.pone.0171499 (PMC5295679; doi:10.1371/journal.pone.0171499)

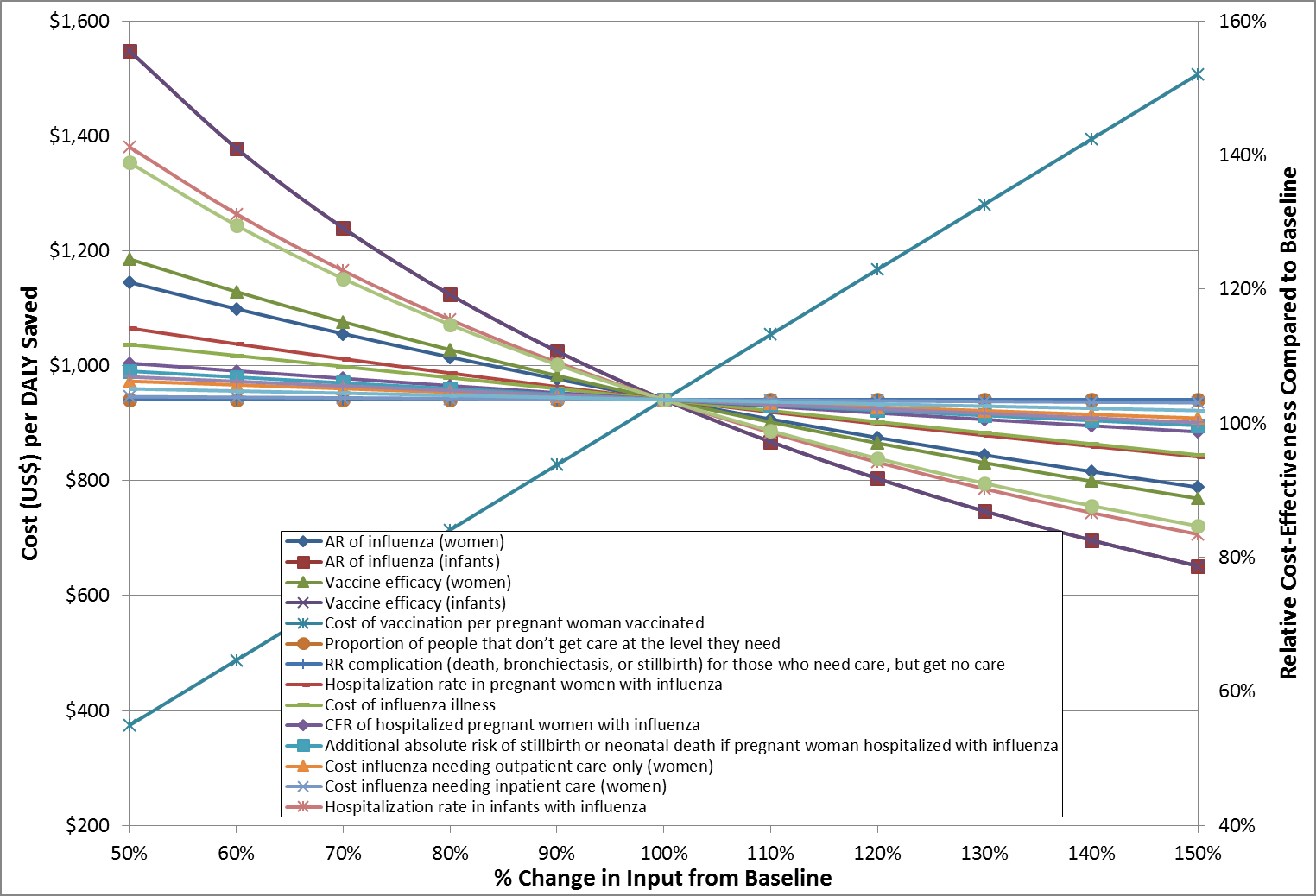

Supplement: S1 Fig — (TIF) [file pone.0171499.s001.tif]
