# Supplementary material for: Cost-effectiveness of maternal influenza immunization in Bamako, Mali: A decision analysis
Source: PLoS One. 2017 Feb 7;12(2):e0171499. doi: 10.1371/journal.pone.0171499 (PMC5295679; doi:10.1371/journal.pone.0171499)
Supplement: S1 Table — (DOCX) [file pone.0171499.s002.docx]

**Supplementary Appendix**

This appendix has been provided by authors to give the readers additional information about their work.

Supplement to: Orenstein EW, et al. Cost-Effectiveness of Maternal Influenza Immunization in Bamako, Mali: A Decision Analysis.

**S1 Table: Complete parameter list with explanations**

| **Parameter** | **Baseline Value** | **Range** | **Distribution** | **References** |
| --- | --- | --- | --- | --- |
| *Burden of influenza illness* |  |  |  |  |
| Attack rate (women) | 0.0096 | 0.0059 – 0.0148 | Triangular | ^1^ |
| Explanation: In the clinical trial of maternal influenza immunization in Mali, there were 40 cases of lab-confirmed influenza (LCI) in 2085 women who received meningococcal conjugate vaccine (MCV) (Table 3 of Tapia et al).^1^ Of these, 19 cases were during pregnancy and 21 cases were post-partum. We averaged these to 20 cases in 2085 women. Using a binomial distribution, this proportion yields a lower confidence limit of 0.59%, a mean estimate of 0.96%, and upper confidence limit of 1.48%. This same attack rate was applied to pregnant women and to post-partum women during the first 6 months of follow-up. | | | | |
| Attack rate (infants) | 0.0284 | 0.0216 – 0.0366 | Triangular | ^1^ |
| Explanation: Due to a precipitous drop in the vaccine efficacy in infants (see below), we looked at infant influenza only in the first 5 months of life. In the intention-to-treat analysis of the clinical trial of maternal influenza immunization in Mali, there were 58 cases of LCI in the first 5 months of life among 2041 infants of mothers vaccinated against MCV. Using a binomial distribution, this proportion yields a lower confidence limit of 2.16%, a mean estimate of 2.84%, and an upper confidence limit of 3.66%. | | | | |
| Risk of hospitalization given influenza (pregnant women) | 0.0064 | 0.0036 – 0.010 | Triangular | ^2–4^ |
| Explanation: There were no hospitalizations of pregnant women infected with LCI during the clinical trial in Mali; however the trial was not powered to assess this parameter with only 19 cases of LCI in pregnant women. Instead, the risk of hospitalization among pregnant women infected with influenza was calculated by multiplying the risk of hospitalization among non-pregnant adults for seasonal influenza in the United States^2^ (4.50, CI: 2.52 -7.29 per 100,000 person-years) by the relative risk of hospitalization for pregnant women compared to non-pregnant women (5.49 in the United States,^3^ 5.38 in Canada,^4^ combined by random-effects meta-analysis weighted by person-years of follow-up) to yield the incidence of influenza-related hospitalization in pregnant women (24.6, per 100,000 pregnant woman-years). This incidence was divided by the baseline attack rate of influenza in pregnant women in the Mali trial (see above – 0.0096 LCI cases per 0.25 pregnant woman-years) to yield the proportion of pregnant women with LCI requiring hospitalization. | | | | |
| Risk of hospitalization given influenza (post-partum women) | 0.0023 | 0.0013 – 0.0038 | Triangular | ^2^ |
| Explanation: There were no hospitalizations of post-partum women infected with LCI during the clinical trial in Mali; however the trial was not powered to assess this parameter with only 21 cases of LCI in post-partum women. Instead, the risk of hospitalization among post-partum women infected with influenza was calculated by dividing the risk of hospitalization among non-pregnant adults for seasonal influenza in the United States^2^ (4.50, CI: 2.52 -7.29 per 100,000 person-years) by the baseline attack rate of influenza in pregnant women in the Mali trial (see above – 0.0091 LCI cases per 0.5 post-partum woman-years) to yield the proportion of post-partum women with LCI requiring hospitalization. | | | | |
| Risk of hospitalization given influenza (infants) | 0.013 | 0.003 – 0.0702 | Triangular | ^1^ |
| Explanation: In the clinical trial of maternal influenza immunization in Mali, there was 1 infant LCI episode requiring hospitalization among 77 cases of LCI in infants of mothers vaccinated with MCV instead of TIV. Using a binomial distribution, this proportion yields a lower confidence limit of 0.03%, a mean estimate of 1.3%, and upper confidence limit of 7.2%. | | | | |
| CFR of influenza-attributable hospitalization (pregnant women) | 0.080 | 0.067 – 0.094 | Triangular | ^5^ |
| Explanation: There were no deaths of pregnant women infected with LCI during the clinical trial in Mali; however the trial was not powered to assess this parameter. Instead, the case-fatality ratio (CFR) of influenza-attributable hospitalization among pregnant women was estimated based on Mosby et al^5^ who reviewed 120 studies following 1625 hospitalized pregnant women in 29 countries infected with 2009 pandemic influenza, 130 of whom died. | | | | |
| CFR of influenza-attributable hospitalization (post-partum women) | 0 | 0 | N/A | ^6^ |
| Explanation: There were no deaths of post-partum women infected with LCI during the clinical trial in Mali; however the trial was not powered to assess this parameter. In a model of hospitalization rates and mortality rates due to influenza in the United Kingdom, the mortality rate in 18-49 year olds was estimated at 0%, so no further CFR was added to our model. | | | | |
| CFR of influenza-attributable hospitalization (infants) | 0.0444 | 0.0119 – 0.0770 | Triangular | ^7,8^ |
| Explanation: There were no deaths of infants infected with LCI during the clinical trial in Mali; however the trial was not powered to assess this parameter. A random-effects meta-analysis of 10 studies of the CFR of severe acute lower respiratory infection (Nair et al^7^ Table 4) in a systematic review of the global burden of influenza in children under 5 years yielded CFR estimate of 2.96% (CI: 0.79% - 5.13%). In a multivariate analysis comparing influenza-attributable CFR among hospitalized infants across different age groups, Cohen et al^8^ found that infants <1 year old had a significant odds ratio of 1.5 times greater risk of mortality. Thus, we multiplied the CFR from Nair et al^7^ by 1.5 to yield a mean estimate of 4.4% (CI: 1.2% - 7.7%) | | | | |
| Additional absolute risk of stillbirth or neonatal death if pregnant woman hospitalized with influenza | 0.0358 | 0.0146 – 0.0876 | Triangular | ^9^ |
| Explanation: While multiple studies have found evidence of improved perinatal outcomes associated with influenza vaccination,^10^ few studies specifically compared differences in perinatal mortality between pregnant women exposed and unexposed to influenza. Fell et al^11^ found 10 studies of fetal death with maternal influenza infection, but found that only 2 were of high quality, 1 of which^12^ was conducted in women with mild-moderate illness, not in women hospitalized with influenza. In the United Kingdom, Pierce et al^9^ found 10 perinatal deaths among 256 infants of women infected with pandemic H1N1 during pregnancy with severe illness, compared to 9 perinatal deaths in 1233 control infants, yielding a risk difference of 3.6% (1.5% - 8.8%). | | | | |
|  |  |  |  |  |
| *Vaccine efficacy* |  |  |  |  |
| Women | 70.3% | 42.2% – 85.8% | Triangular | ^1^ |
| Explanation: In the clinical trial of maternal influenza immunization in Mali, overall vaccine efficacy among women was 70.3% (CI: 42.2% - 85.8%). This same value was used for both pregnant and post-partum women. | | | | |
| Infants in 1^st^ 5 months of life | 57.3% | 30.6% – 74.4% | Triangular | ^1^ |
| Explanation: In the clinical trial of maternal influenza immunization in Mali, the vaccine efficacy to prevent influenza in infants of vaccinated mothers was 33.1% (CI 3.7% – 53.9%) by ITT analysis and was 37.3% (CI 7.6% – 57.8%) among infants born >14 days among all infants. However, the vaccine efficacy dropped off precipitously after the first 5 months of life, most likely due to loss of maternal antibodies transmitted through the placenta after maternal immunization. We therefore decided to focus on the protection afforded during the first 5 months of life (see Tapia et al^1^ Table 2), yielding vaccine efficacy of 57.3% (CI: 30.6% – 74.4%). | | | | |
|  |  |  |  |  |
| *Utilities* |  |  |  |  |
| DALYs lost for maternal death | 32.27 | --- | Point estimate | ^13^ |
| Explanation: The number of disability adjusted life years lost for a maternal death is calculated as the average of life expectancy for females in Mali from the Global Burden of Diseases in 2013^13^ minus the average age at delivery during the clinical trial in Mali (24.7).^1^ GBD 2013 defines DALYs as years of life lost (YLL) plus years lived with disability (YLD) and does not time-discount or age-weight these estimates.^14^ Of note, given that current life expectancy in Mali is substantially lower than average, we chose not to use the World Health Organization Standard Expected Years of Life Lost (which would have set this parameter at 67.24), which are based on projected frontier period life expectancy and life table for the year 2050. | | | | |
| DALYs lost for infant death or stillbirth | 57.275 | --- | Point estimate | ^13^ |
| Explanation: The number of disability adjusted life years lost for a neonatal death is calculated as the average of life expectancy for males and females in Mali from GBD in 2013. GBD 2013 defines DALYs as years of life lost (YLL) plus years lived with disability (YLD) and does not time-discount or age-weight these estimates.^13^ Of note, given that current life expectancy in Mali is substantially lower than average, we chose not to use the World Health Organization Standard Expected Years of Life Lost (which would have set this parameter at 91.94), which are based on projected frontier period life expectancy and life table for the year 2050. | | | | |
|  |  |  |  |  |
| *Access to Care* |  |  |  |  |
| Proportion with inadequate access to care | 0.604 | 0.507 – 0.730 | Triangular | ^15^ |
| Explanation: The proportion of mothers and infants without access to care was taken from the Demographic and Health Survey conducted in Mali in 2012.^15^ In this survey, only 60.4% of participants who had been sick in the last 30 days did not seek care in a public or private health center. The lower limit was based on the richest quintile (of whom 50.7% did not seek care), and the upper limit based on the poorest quintile (of whom 73.0% did not seek care). | | | | |
| Relative risk of death without access to care | 3 | 1 – 5 | Triangular | Assumption |
|  |  |  |  |  |
| *Costs of Illness* |  |  |  |  |
| Outpatient influenza episode (women) | 4.83 | 3.72 – 6.27 | Triangular | Present study |
| Explanation: In the present study conducted over the course of the clinical trial in Mali, we captured the costs of 52 outpatient LCI episodes in women (23 of 24 total LCI episodes in pregnant women and 29 of 29 total episodes in post-partum women). These costs were modeled with a log-normal distribution, yielding average cost of US$4.83 (CI: $3.72 - $6.27). There was no significant difference between episodes in women vaccinated with TIV ($6.37, CI: $3.02 - $13.46) compared to MCV ($4.36, CI: $3.44 - $5.96), so all episodes were included when estimating this parameter. | | | | |
| Outpatient influenza episode (infants) | 4.41 | 3.99 – 4.87 | Triangular | Present study |
| Explanation: In the present study, we captured the costs of 132 outpatient LCI episodes in infants out of 140 total episodes (these include 129 first episodes of LCI as reported in Tapia et al[Tapia***] as well as 11 repeat episodes in the same infant across 2 years of surveillance). These costs were modeled with a log-normal distribution, yielding average cost of US$4.41 ($3.99 - $4.87). There was no significant difference between episodes in infants vaccinated with TIV ($3.99, CI: $3.45 - $4.62) compared to MCV ($4.70, CI: $4.11 - $5.37), so all episodes were included when estimating this parameter. | | | | |
| Inpatient influenza (women) | 157.50 | 34.39 – 280.62 | Triangular | Present study |
| Explanation: In the present study, there were no pregnant or post-partum women hospitalized for LCI. However, 2 women were hospitalized for influenza-like illness (ILI), with total costs of $34.39 and $280.62. Given the paucity of LCI hospitalization data and the absence of significant differences in cost between ILI and LCI episodes overall (see Table 2 in the main text), we used the average of these two episodes as the mean estimate, with lower and upper limits equal to the two values. | | | | |
| Inpatient influenza episode (infants) | 157.50 | 131.20 – 189.09 | Triangular | Present study |
| Explanation: In the present study, there was only 1 infant hospitalized with LCI (with cost of $247.37), but 63 total ILI hospitalization episodes were captured. Using log-normal distribution, the average cost of an ILI hospitalization was $157.50 (CI: $131.20 – $189.09). | | | | |
| *Costs of Vaccination Program* |  |  |  |  |
| Cost per pregnant woman vaccinated | $1.00 | $0.50 - $2.00 | Triangular | ^16^ |
| Explanation: In Griffiths et al,^16^ maternal tetanus vaccines were given for a total programmatic cost of $0.41 per dose given or $1.19 for complete vaccination. In this study, the cost of vaccine itself was $0.04. As influenza vaccine may prove more expensive, we have assumed a higher total cost per pregnant woman vaccinated of $1.00 with lower limit $0.50 and upper limit $2.00. | | | | |

# References:

1 Tapia MD, Sow SO, Tamboura B, *et al.* Maternal immunisation with trivalent inactivated influenza vaccine for prevention of influenza in infants in Mali: a prospective, active-controlled, observer-blind, randomised phase 4 trial. *Lancet Infect Dis* 2016; published online May. DOI:10.1016/S1473-3099(16)30054-8.

2 Dao CN, Kamimoto L, Nowell M, *et al.* Adult Hospitalizations for Laboratory‐Positive Influenza during the 2005–2006 through 2007–2008 Seasons in the United States. *J Infect Dis* 2010; **202**: 881–8.

3 Neuzil KM, Reed GW, Mitchel EF, Simonsen L, Griffin MR. Impact of influenza on acute cardiopulmonary hospitalizations in pregnant women. *Am J Epidemiol* 1998; **148**: 1094–1102.

4 Dodds L, McNeil SA, Fell DB, *et al.* Impact of influenza exposure on rates of hospital admissions and physician visits because of respiratory illness among pregnant women. *Can Med Assoc J* 2007; **176**: 463–8.

5 Mosby LG, Rasmussen SA, Jamieson DJ. 2009 pandemic influenza A (H1N1) in pregnancy: a systematic review of the literature. *Am J Obstet Gynecol* 2011; **205**: 10–8.

6 Matias G, Taylor RJ, Haguinet F, Schuck-Paim C, Lustig RL, Fleming DM. Modelling estimates of age-specific influenza-related hospitalisation and mortality in the United Kingdom. *BMC Public Health* 2016; **16**. DOI:10.1186/s12889-016-3128-4.

7 Nair H, Brooks WA, Katz M, *et al.* Global burden of respiratory infections due to seasonal influenza in young children: a systematic review and meta-analysis. *The Lancet* 2011; **378**: 1917–1930.

8 Cohen C, Moyes J, Tempia S, *et al.* Mortality amongst patients with influenza-associated severe acute respiratory illness, South Africa, 2009-2013. *PloS One* 2015; **10**: e0118884.

9 Pierce M, Kurinczuk JJ, Spark P, Brocklehurst P, Knight M, on behalf of UKOSS. Perinatal outcomes after maternal 2009/H1N1 infection: national cohort study. *BMJ* 2011; **342**: d3214–d3214.

10 Bratton KN, Wardle MT, Orenstein WA, Omer SB. Maternal Influenza Immunization and Birth Outcomes of Stillbirth and Spontaneous Abortion: A Systematic Review and Meta-analysis. *Clin Infect Dis* 2015; **60**: e11–9.

11 Fell D, Savitz D, Kramer M, *et al.* Maternal influenza and birth outcomes: systematic review of comparative studies. *BJOG Int J Obstet Gynaecol* 2016; published online May. DOI:10.1111/1471-0528.14143.

12 Håberg SE, Trogstad L, Gunnes N, *et al.* Risk of Fetal Death after Pandemic Influenza Virus Infection or Vaccination. *N Engl J Med* 2013; **368**: 333–40.

13 Murray CJ, Barber RM, Foreman KJ, *et al.* Global, regional, and national disability-adjusted life years (DALYs) for 306 diseases and injuries and healthy life expectancy (HALE) for 188 countries, 1990–2013: quantifying the epidemiological transition. *The Lancet* 2015; **386**: 2145–2191.

14 Mathers CD, Stevens G. WHO methods and data sources for global burden of disease estimates 2000-2011. Geneva: World Health Organization, 2013 http://www.who.int/healthinfo/statistics/GlobalDALYmethods_2000_2011.pdf?ua=1 (accessed Jan 31, 2016).

15 Cellule de Planification et de Statistiques (CPS/SSDSPF), Institut National de la Statistique (INSTAT/MPATP), INFO-STAT, ICF International. Enquête Démographique et de Santé au Mali 2012-2013. Rockville, Maryland, USA, 2014 http://dhsprogram.com/pubs/pdf/FR286/FR286.pdf (accessed Jan 30, 2016).

16 Griffiths UK, Wolfson LJ, Quddus A, Younus M, Hafiz RA. Incremental cost-effectiveness of supplementary immunization activities to prevent neonatal tetanus in Pakistan. *Bull World Health Organ* 2004; **82**: 643–651.
